# PANoptosis-like death in acute-on-chronic liver failure injury

**DOI:** 10.1038/s41598-023-50720-1

**Published:** 2024-01-03

**Authors:** Qianling Ye, Hanjing Wang, Yue Chen, Yihao Zheng, Yuqiong Du, Chongyang Ma, Qiuyun Zhang

**Affiliations:** 1 School of Traditional Chinese Medicine, Capital Medical University, Beijing, 100069 China; 2 Beijing Key Laboratory of TCM Collateral Disease Theory Research, Beijing, 100069 China

**Keywords:** Cardiology, Medical research, Molecular medicine, Pathogenesis

## Abstract

The pathogenesis of Acute-on-chronic liver failure (ACLF) involves several forms of cell death, such as pyroptosis, apoptosis, and necroptosis, which consist of PANoptosis. To explore PANoptosis as a regulated cell death pathway in ACLF. Firstly, a bioinformatic strategy was used to observe the role of the PANoptosis pathway in ACLF and identify differentially expressed genes related to PANoptosis. Enrichment analysis showed that PANoptosis-related pathways were up-regulated in ACLF. We screened out BAX from the intersection of pyroptosis, apoptosis, necroptosis, and DEGs. Secondly, we screened articles from literature databases related to PANoptosis and liver failure, and specific forms of PANoptosis were reported in different experimental models in vitro and in vivo. Secondly, we established a model of ACLF using carbon tetrachloride-induced liver fibrosis, followed by D-galactosamine and lipopolysaccharide joint acute attacks. A substantial release of inflammatory factors(IL-6, IL-18, TNFα, and IFNγ) and the key proteins of PANoptosis (NLRP3, CASP1, GSDMD, BAX, CASP8, CASP3, CASP7, and MLKL) were detected independently in the ACLF rats. Finally, we found that combining TNF-α/INF-γ inflammatory cytokines could induce L02 cells PANoptosis. Our study highlighted the potential role of ACLF and helps drug discovery targeting PANoptosis in the future.

## Introduction

Acute-on-chronic liver failure (ACLF) is a syndrome that occurs in patients with acute decompensation cirrhosis, characterized by high short-term mortality because of different combinations of organ failure^1^. Several researchers are actively investigating the pathogenesis of ACLF to develop new effective therapeutic methods. The development of ACLF is a complex and multifactorial process that is yet to be fully elucidated. Several factors and pathways interact and contribute to the progression of liver injury and subsequent organ failure, and two theories suggest the underlying mechanisms of ACLF. According to the first theory, an acute trigger activates immune cells and inflammatory cytokine pathways, leading to hepatocyte damage and necrosis. The second theory, also known as the “triple whammy” theory, suggests that patients with viral ACLF sequentially experience immune-mediated damage, ischemic and hypoxic damage, and endotoxemia. Notably, both theories suggest that an imbalance in the immune system and an inflammatory response are critical for causing liver injury in ACLF^2,3^. Injured or dying liver cells release damage-associated molecular patterns (DAMPs), and recognition of these DAMPs by specific pattern-recognition receptors (PRRs) in the host exacerbates the inflammatory response, resulting in the release of pro-inflammatory factors that trigger and worsen the inflammatory response, inflicting further damage to liver tissue. The combination of high concentrations of pathogen-associated molecular patterns (PAMPs; released by microbes) and DAMPs can trigger an increased systemic inflammatory response, leading to the release of several pro-inflammatory cytokines. This initiates a cytokine storm, resulting in hepatocellular damage, necrosis, and extrahepatic tissue damage, which can evolve into liver failure or even extrahepatic organ failure^3,4^.

Systemic inflammation is the main pathologic feature of ACLF. When ACLF occurs, many hepatocytes enter into a state of pathologically active or passive death. The ACLF pathogenesis involves several forms of cell death, such as necrosis^5^, apoptosis^6^, necroptosis^7^, and pyroptosis^7^. Hepatocyte necroptosis is a regulated process morphologically similar to necrosis and is accompanied by abnormal mitochondrial function, cell membrane rupture, organelle swelling, and inflammatory responses^8^. Hepatocyte scorch death is a newly discovered form of cell death that is characterized by chromatin DNA breakage, cytoplasmic swelling, multiple vesicle-like bulges on the cell surface (leading to uneven membrane rupture and death), and a persistent and exaggerated immune inflammatory amplification^9^. Hepatocyte apoptosis is characterized by blistering of the cell membrane, cell shrinkage, nuclear fragmentation, chromatin condensation, chromosomal DNA fragmentation, and the formation of apoptotic vesicles. Its activation pathway contributes to the caspase (cysteine protease)-activation cascade downstream of mitochondrial cytochrome c release^10^. The three programmed cell death pathways, namely pyroptosis, apoptosis, and necroptosis, occurring in hepatocytes show crosstalk and co-regulation and can switch among themselves under certain conditions. For example, receptor-interacting serine/threonine protein kinase (RIPK)3 activity increased following the inhibition of caspase8 (CASP8), and cells underwent necroptosis. Similarly, RIPK3 inhibition enhanced the apoptotic activity of CASP8. CASP8 and Fas-associated death domain (FADD) regulate classical and non-classical NOD-like receptor family, pyrin domain-containing 3 (NLRP3) inflammasomes, suggesting a link between apoptosis and cell scorching. In addition, the apoptotic cysteine proteases, CASP3 and CASP7, specifically block cell scorching by cleaving the gasdermin D (GSDMD) at a site different from that of inflammatory cysteine^11^. A hyperactive immune system may drive the rapid early dominant hepatocyte death^12^. Cellular dysfunction and death caused by excessive inflammatory responses are often mediated by pro-inflammatory cytokines, such as tumor necrosis factor-alpha (TNFα), interleukin (IL)-1β, IL-6, and interferon-gamma (IFNγ)^13,14^. High production of TNFα, IL-1β, IL-12, IL-17, sST2, and sTRAIL was associated with the identification of a large number of apoptotic cells. These cells were induced by molecules, such as TRADD, RIPK1, RIPK3, caspase-8, caspase-3, and caspase-7^15^. The release of IFN-γ promoted the phosphorylation of RIPK1, RIPK3, and mixed lineage kinase domain-like (MLKL) to induce necroptosis^16^. Inflammatory vesicles containing molecules, such as IL1α, IL33, and HMGB1, can activate sensor proteins including NLRC4, NLRP1, AIM2, pyrin, and NLRP3, resulting in caspase-1 activation to induce pyroptosis^17^.

PANoptosis (Pyroptosis, Apoptosis, and Necroptosis) is a unique inflammatory regulated cell death (RCD) regulated by the PANoptosome^18^. When PRRs recognize PAMPs expressed by microbes and DAMPs released after aseptic injury, they bind to inflammatory factors and transduce signals through receptors containing death domains to initiate a highly interconnected process of cell death called PANoptosis. The process involves the interaction and activation of inflammatory vesicles, pyroptosis, apoptosis, and programmed necrosis, and it cannot be blocked by the end effectors of any individual pathway^19^. PANoptosome is similar to the inflammatory vesicle complex; they share a core protein that promotes the activation of downstream cell death effector molecules. Downstream cytokines (e.g., IL-1β, IL-18, IL-6, TNFα, and IFNγ) and DAMPs act as alarm proteins to initiate and enhance the inflammatory response^20^. PANoptosome promotes FADD–CASP8-dependent apoptosis and necroptosis and activates the pyrin domain in NLRP3 inflammasome via RIPK3-mediated phosphorylation of MLKL for initiating pyroptosis^21^. PANoptosis is involved in several other diseases, such as sepsis, systemic inflammatory diseases, multiple organ failure, cancer, novel coronavirus infection, and cytokine shock in addition to infectious diseases^22–24^. These diseases involve severe systemic inflammatory syndromes with elevated levels of circulating pro-inflammatory cytokines or cytokine storms in the patient^22^.

A detailed understanding of the molecular basis of PANoptosis is crucial for regulating systemic inflammatory and immune responses and developing targeted inhibitors and activators for treating inflammatory cell death. ACLF is characterized by intense systemic inflammation, inflammatory cell infiltration, and the release of inflammatory factors, leading to massive necrosis of hepatocytes. Many hepatocytes enter pathologic PANoptosis, which plays an essential role in cell death and inflammatory factor storm. PANoptosome can fundamentally integrate cell death patterns and drive this powerful form of inflammatory cell death in pathologic situations. The role of PANoptosis in ACLF is an active area of research. However, the exact mechanisms and the extent to which PANoptosis-like cell death occurs in ACLF are unknown. Therefore, further studies are needed to elucidate the specific molecular mechanisms and potential therapeutic targets associated with this type of cell death. Here, we conducted bioinformatic analyses, literature mining, and animal experiments to explore the role of PANoptosis in ACLF.

## Materials and methods

### Gene expression spectrum data acquiring and processing

We downloaded the dataset GSE139602 in the GEO database (http://www.ncbi.nlm.nih.gov/geo). GSE139602^25^ includes transcriptome data of liver biopsies performed on 6 healthy liver biopsies, 5 eCLD liver biopsies, 8 compensated cirrhosis liver biopsies, 12 decompensated cirrhosis liver biopsies, and 8 acute—chronic liver failure liver biopsies based on the GPL13667 platform [HG-U219] Affymetrix Human Genome U219 array. We downloaded the biochip data in the MINIML package, which consists of expression spectrum datasets stored as family files, from the GEO database and imported it into the Sangerbox software (http://sangerbox.com/Tool)^26^. The downloaded data were processed in the GEO converter to match the probe ID data to the gene symbol. When multiple probes matched a gene, it was replaced by the median to further obtain the gene sample information and expression matrix of the expression profile data set. The gene expression matrix was further standardized to eliminate sample differences. The quantiles of the gene expression matrix were standardized using the data processing tool in the Sangerbox software to obtain the standardized gene expression matrix.

### Differentially expressed genes (DEGs) filtering

The “Limma” package 3.26.8 was used for this analysis^27^. Log2FC represented the logarithm of base 2 of the fold change (FC). The false discovery rate (FDR) was obtained by correcting the *P*-values of the different significance values. The screening criteria were set to |logFC|> 1 and FDR < 0.05.

### Enrichment analysis

Gene Set Enrichment Analysis (GSEA)^28^ is a calculation method used to determine whether a predefined gene set shows statistically significant and consistent differences between two biological states (such as phenotypes). The standardized gene expression matrix and sample grouping files were imported into the GSEA simple analysis tool of the Sangerbox software. Kyoto Encyclopedia of Genes and Genomes (KEGG) was selected as the reference database, and the REACTOME database was selected for analysis. REACTOME calculates the enrichment score, normalized enrichment score (NES), and normalized significance level and corrects multiple hypothesis tests according to the algorithm. The data sources were set as Gene Ontology (GO) and KEGG, and the analysis standard was set as adjusted *P* < 0.05.

### Immune cell infiltration and protein–protein interaction (PPI) network analyses

We used the R language software package to compare and calculate the infiltration of immune cells in each sample according to CIBERSORT (https://cibersort.stanford.edu)^29^. The samples were selected based on the significance threshold of *P* < 0.05, and the percentage of each immune cell in the selected samples was calculated. The DEGs were uploaded into the STRING online database, and the obtained text files were imported into the Cytoscape 3.8.0 software. The "mcode" plugin in Cytoscape was used to filter out subnetworks from the DEG data.

### Source of data collection

**Figure 1:**
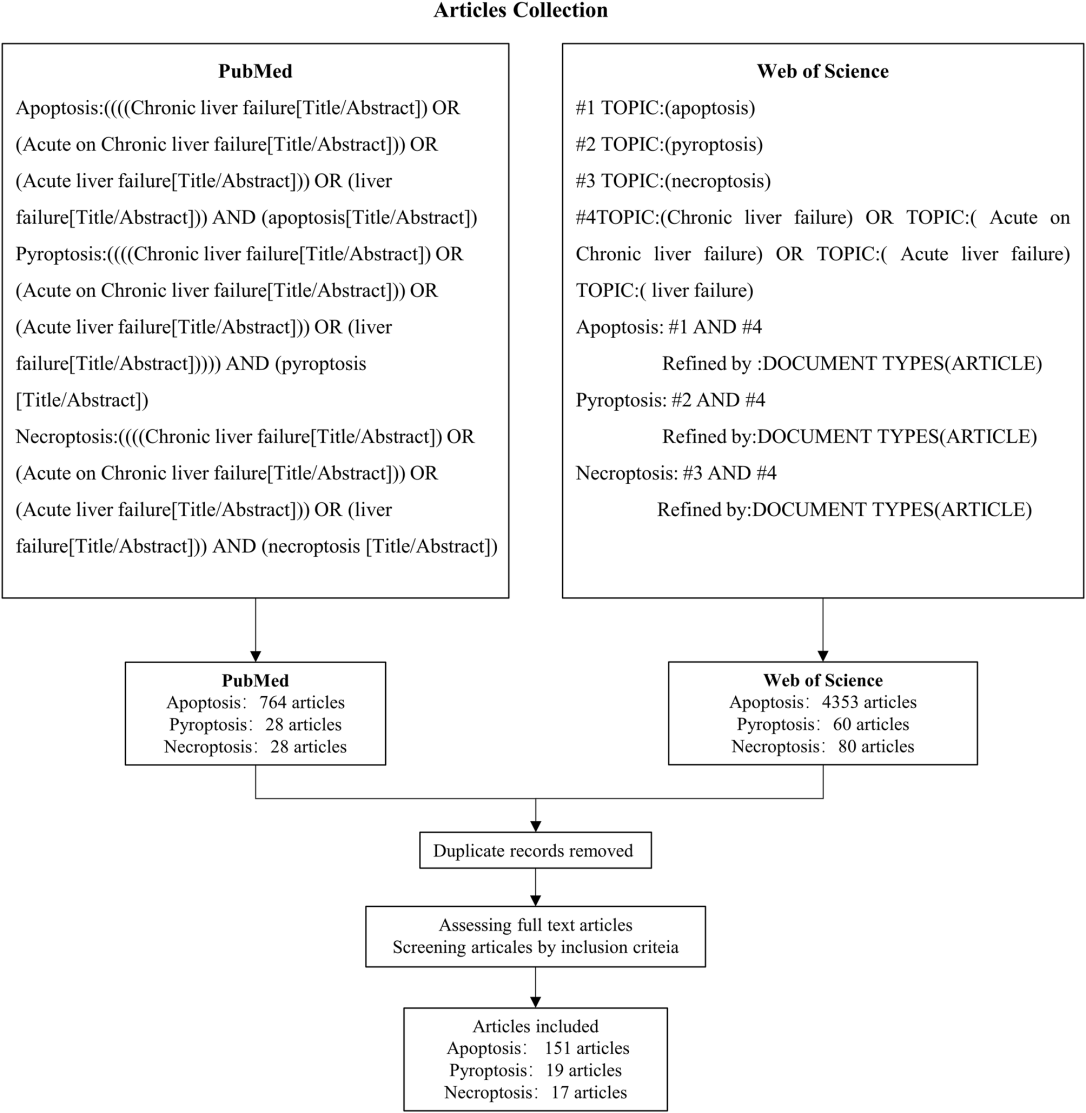
Flow chart of articles screening.

We chose PubMed and Web of Science databases as data sources. Keywords were divided into two groups: (a) regulated cell death (RCD) including pyroptosis, apoptosis, and necroptosis and (b) liver failure and its MeSH apposition, hyponym, or supernym. We used the refining function of each database to limit the search field to hepatobiliary medicine, hepatobiliary surgery, or digestive medicine. Article types were limited to research articles. The final retrieval date of the published time range of the retrieved literature was December 9, 2022. The retrieval strategy of each database was customized according to the database standards and the scale of the retrieval document. The inclusion criteria were supplemented by manually searching the references of the research articles included in this study. The articles retrieved from each database were combined according to the three forms of cell death, and the duplicate documents were screened and deleted according to the inclusion criteria. Figure 1 shows the methods and processes used for data collection.

### Literature inclusion and exclusion criteria

The articles were selected based on the following criteria: (1) The core objective of the paper was to research liver failure or animal or cell models that can represent liver failure. (2) Rodents, primary cells, or passage cell lines were used as experimental materials. (3) The target organ/cells damaged in the study could be the liver, primary hepatocytes, or subcultured cells. (4) The experimental results included two or more test results to indicate the existence of pyroptosis, apoptosis, or necroptosis, and at least one test detected a key protein of any RCD. (5) The experimental design included model and control groups. The exclusion criteria for the research articles were: (1) Target cells of the experimental study were not hepatocytes (such as hepatic stellate cells, macrophages, endothelial cells, and human primary monocytes). (2) The process and standard of model establishment were not described. (3) The experimental evidence for the existence of any of the three forms of cell death was insufficient.

### Data collating, mining, and classifying analysis

Data on patients with liver failure, cell types, animal species, modeling methods, cell death evaluation, and representative molecular detection results of different cell death modes were extracted from the included literature. Further, we extracted the experimental data to evaluate pyroptosis, apoptosis, and necroptosis and ensured two conditions should be met to determine whether liver failure in the experiment induced pyroptosis, apoptosis, or necroptosis. First, the researchers used standard detection methods, such as propidium iodide staining, terminal deoxynucleotidyl transferase-mediated dUTP nick-end labeling assay, flow cytometry, cell counting kit-8 assay, and lactate dehydrogenase assay, to evaluate the degree of cell death induced by liver failure. Second, the authors reported the detection of at least two or more key proteins/genes of pyroptosis, apoptosis, and necroptosis (Table 1)^30–33^. Further, the results of the experimental group should be statistically significant compared with that of the control group. The document items exported from the database were imported into the document management software, and two researchers of this specialty independently read the documents, conducted article selection and data mining, and obtained relevant data from the documents. Then, they compared the data obtained and summarized the consistent results in a table. If differences were observed, they discussed or submitted the relevant information to the third researcher for assistance. The cluster analysis of the in vitro experiments was based on the cell type used to study pyroptosis, apoptosis, and necroptosis of cells. Cluster analysis was carried out to group the animals based on their characteristics or other relevant factors to ensure that the animals used in the in vivo experiments had similar baseline characteristics before the liver failure operations. The core data obtained were collated and analyzed using the EndNote 20 and Microsoft Excel 2019 softwares.

**Table 1:** The key proteins of PANoptosis.

Cell death type	Key proteins
Pyroptosis	TLR4, NLRP3, ASC, CASP-1, CASP-4, CASP-6, CASP-1, CASP-11, C-CASP-1, GSDMD, GSDMD-N, IL-1β, IL-18
Apoptosis	CASP-3, CASP-7, CASP-8, CASP-9, C-CASP-3, C-CASP-7,C-CASP-8,Bcl-2, BAX
Necroptosis	RIP1, p-RIP1, RIP3, p-RIP3, MLKL, p-MLKL

### Animals

Male Wistar rats (n = 20) weighing 180–200 g were housed in a specific-pathogen-free environment. The animals were provided free access to water and food. The room temperature was 22.4 ± 3.0 ℃, relative humidity was 51.3 ± 2.2%, and the light/dark cycle was 12-h/12-h. Rats were purchased from Beijing Vital River Laboratory Animal Technology Co., Ltd., Beijing, China.

### Establishing the ACLF model

Wister rats were randomly divided into two groups, namely the normal group (Normal; n = 9) and the ACLF model group (ACLF; n = 11). The ACLF group rats were injected with 40% carbon tetrachloride (CCL4) and olive oil solution through their abdominal cavity for 10 weeks to establish the cirrhosis model. Then, d-galactosamine(D-GalN) (500 mg/kg) and lipopolysaccharide (LPS; 100 μg/kg) were intraperitoneally injected into these rats to induce the acute attack and establish the ACLF model^34^. The same volume of 0.9% physiologic saline was administered to rats in the normal group at each time point.

### Histopathologic analysis

We fixed the liver tissues in 10% formalin for at least 48 h, embedded it in paraffin, and prepared paraffin sections. The sections were stained with hematoxylin & eosin (HE) and Masson’s trichome and scanned using the Pannoramic SCAN II slide scanner (3DHISTECH Ltd., Budapest, Hungary). The CaseViewer software was used to obtain images and detect the pathologic damage in the liver tissues from each group.

### Biochemical analysis

Blood samples were collected from the rats in both groups; the samples were kept at 25 °C for 4 h and centrifuged at 825 g for 15 min at 4 °C to obtain the serum samples. The concentrations of serum alanine aminotransferase (ALT), aspartate aminotransferase (AST), total bilirubin (TBIL), albumin (ALB), and creatinine (CRE) were quantified in each sample using an automatic biochemical analyzer (Rayto, Chemray-240).

### Enzyme-linked immunosorbent assay (ELISA)

ELISA kits were used to determine the expression levels of IL-6 (CUSABIO, CSB-E04640r), IL-1β (Servicebio, GER0002), IFN-γ (BOSTER, EK0374), and TNFα (BOSTER, EK0526) in the serum samples. All experiments were carried out according to the manufacturer’s instructions.

### Western blotting

We took approximately 43–50 mg liver tissue from each group, added RIPA lysate at the ratio of 1:7, homogenized the sample, and incubated it on ice for 1 h. Then, the homogenized samples were centrifuged at 4 ℃ and 13,201 g/min for 20 min. The supernatant was collected in separate tubes, and the protein concentration in each supernatant sample was quantified using the BCA protein quantitative test kit according to the manufacturer’s instructions. The proteins were separated using electrophoresis and subsequently transferred onto the PVDF membranes. The membranes were incubated with 5% skimmed milk at room temperature for 2 h to block the unoccupied binding sites. Next, the membranes were incubated with diluted solutions of anti-caspase-1 (CST, #83,383, 1:1000), anti-caspase-8 (CST, #4790, 1:1000), anti-MLKL (ABclonal, A13452, 1:1000), anti-phospho-MLKL (ThermoFisher, #PA5-105678, 1:2000), anti-caspase-3 (CST, #9662, 1:1000), anti-caspase-7 (CST, #9492, 1:1000), anti-NLRP3 (Abcam, ab263899, 1:1000), anti-GSDMD (Abcam, ab219800, 1:1000), anti-GSDMD-N (Sigma, SAB2108448, 1:800), anti-BAX (Boster, A00183, 1:1000), or anti-GAPDH (LabLead, G0100, 1:5000) antibodies at 4 °C overnight. Next, the membranes were washed using the appropriate buffer and incubated with corresponding secondary antibodies, including donkey anti-mouse IgG (LabLead, S0100, 1:5000) and goat anti-rabbit IgG (LabLead, S0101, 1:5000), at room temperature for 1 h. Finally, the membranes were developed using the Vilber FUSION FX6 XT gel chemiluminescence imaging analysis system (Vilber Lourmat, Marne La Vallee, France), and the images were analyzed using ImageJ v1.8.0.

### Cell culture and treatment

Cell Culture and Treatment Normal human hepatocytes (L02 cells) obtained from the Cell Bank of Type Culture Collection of the Chinese Academy of Sciences (Shanghai, China) were maintained in DMEM media (gibco, America, cat: 8,123,435) supplemented with 10% (v/v) fetal bovine serum (FBS) (Analysis Quiz, China, cat: AQ-MV-09900), streptomycin at 37 °C in a humidified atmosphere with 5% CO2. The JDNW stock solution was diluted with culture media immediately before the experiment. The Que stock solution was prepared in DMSO and diluted with culture media immediately before the experiment.

### Cell viability assay

The effects of JDNW and Que on the viability of L02 cells were evaluated and counted using a Cell Counting Kit-8 (CCK-8) assay (New Cell and Molecular Biotech, China; cat: C6005), according to the manufacturer’s instructions. Briefly, cells were grown on 96-well plates at a density of 1 × 104 for 24 h. After treatment with JDNW and Que and TNFα (Preprotech, America, cat: 300–01 A)/IFN γ (Preprotech, America, cat: 300-02) for the indicated time, the cells were incubated with 10 ml of the CCK-8 solution. After incubation at 37 °C for 1 h in a humidified CO2 incubator, the absorbance was monitored at 450 nm on a microplate reader (Thermo Scientific, USA). The cell viability was calculated by comparing the optical densities of samples to the control (media only) cells. The optical density of the formazan formed in control cells was 100% viability.

### Propidium iodide nucleic acid stain assay

Set up 6-well assay plates containing cells in a culture medium. Add test compounds and vehicle controls to appropriate wells so the final volume is 2 ml in each well. Incubate cells at 37 °C for the desired test exposure period. Cells were treated with the indicated cytokines and stained with propidium iodide (PI; Life Technologies, P3566) following the manufacturer’s protocol. The plate was scanned by fluorescence microscope (Japan, Olympus IX83), and fluorescent and phase-contrast images (4 image fields/well) were acquired in real-time every 1 h from 0 to 72 h post-treatment. PI-positive dead cells are marked with a red mask for visualization.

### Statistical analyses

The SPSS 21.0 software was used to process and analyze the experimental data, and the measurement data. If the data of each group did not conform to the normal distribution, the analysis of variance and t-test were used to compare groups. *P*-value < 0.05 was considered statistically significant.

### Ethical approval

All animal experiments were performed according to ARRIVE guidelines and in the Experimental Animal Center of Capital Medical University (Beijing, China). All protocols were carried out according to the current legislation relating to animals and experiments involving animals and were approved by the Animal Experiments and Experimental Animal Welfare Committee of Capital Medical University (approval ID: AEEI-2022-021).

## Results

### Screening of DEGs

**Figure 2:**
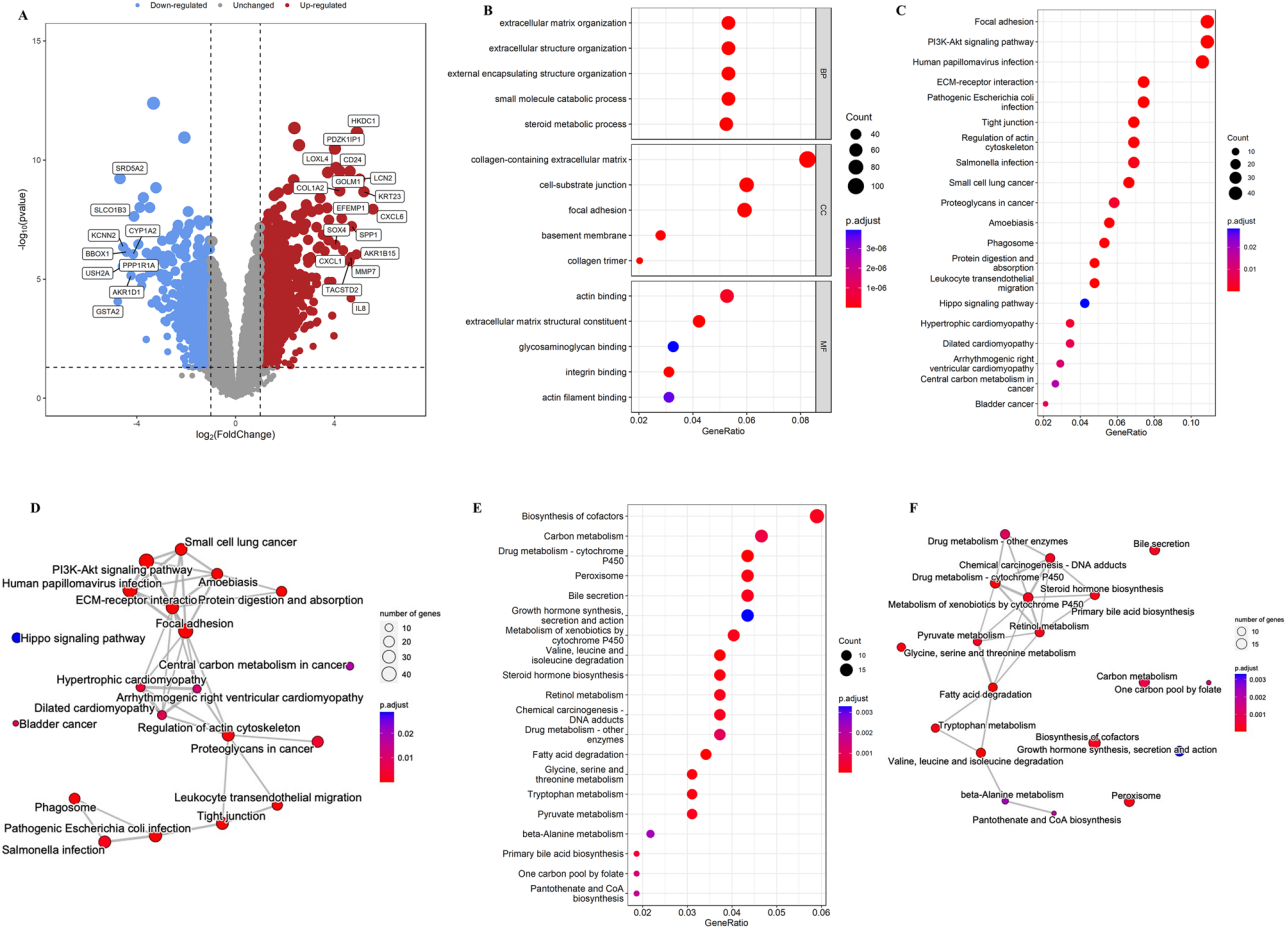
Screening of DEGs in GSE139602 and enrichment analysis. (**A**) Differentially expressed genes (DEGs) are presented in a volcano plot. Blue plots represent downregulated DEGs, and red plots represent upregulated DEGs. (**B**) The GO enrichment bubble chart of the top 5 BPs, MFs, and CCs. (C-D) The top 20 KEGG pathways of upregulated DEGs. (**E**–**F**) The top 20 KEGG pathways of downregulated DEGs.

Our screening criteria of |log_2_FC |> 1 and FDR < 0.05 resulted in 1446 DEGs in the ACLF and normal control groups of GSE139602, including 829 upregulated and 617 downregulated DEGs. Figure 2A shows the volcano plot for the visualization of DEGs.

### Pathway enrichment analysis and functional annotation

We performed a GO enrichment analysis to classify the functions of DEGs. The results showed that the biological processes of ACLF mainly included extracellular matrix organization, extracellular structure organization, external encapsulating structure organization, small molecule catabolic process, and steroid metabolic process. The cellular components of ACLF mainly included a collagen-containing extracellular matrix, cell-substrate junction, focal adhesion, basement membrane, and collagen trimer. The molecular functions of ACLF mainly included actin binding, extracellular matrix structural constituent, glycosaminoglycan binding, integrin binding, and actin filament binding (Fig. 2B). The upregulated DEGs were mainly engaged in focal adhesion, PI3K-Akt signaling pathway, human papillomavirus infection, extracellular matrix–receptor interaction, and pathogenic *Escherichia coli* infection (Fig. 2C, D). Downregulated DEGs were mainly engaged in the biosynthesis of cofactors, carbon metabolism, drug metabolism-cytochrome P450, and peroxisome bile secretion (Fig. 2E, F).

### Functional annotation of GSE139602

**Figure 3:**
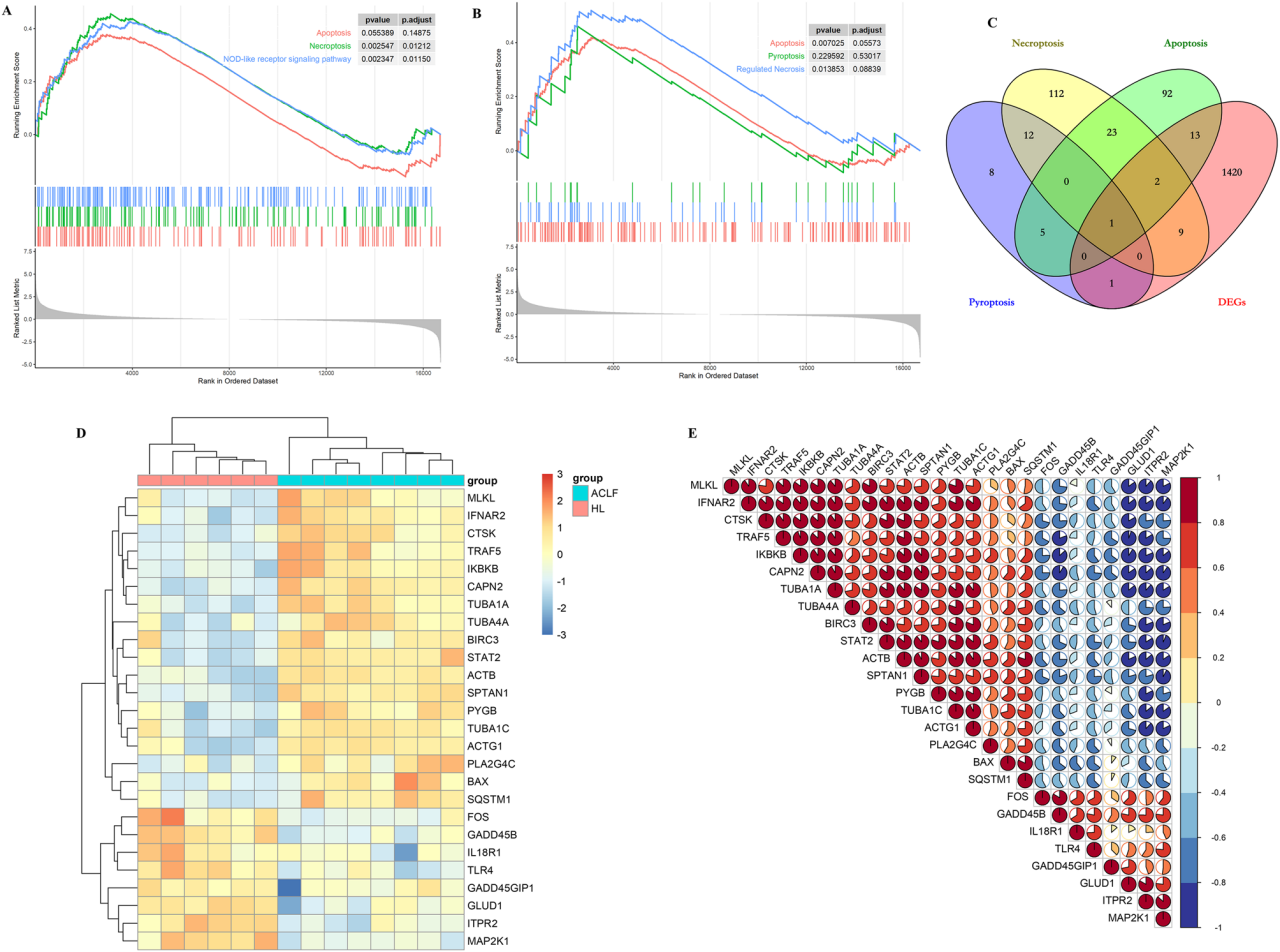
Functional annotation of GSE139602. (**A**) Significantly enriched gene sets of patients with ACLF in GSE139602 obtained through GSEA from KEGG. (**B**) Significantly enriched gene sets of patients with ACLF in GSE139602 were obtained through GSEA from REACTOME. (**C**) Venn diagram shows 1446 DEGs overlapping with pyroptosis, apoptosis, and necroptosis. (**D**) Heat map shows significantly enriched genes; yellow represents high expression, and blue represents low expression. (**E**) Correlation analysis of significantly enriched genes; red represents a positive correlation, and blue represents a negative correlation.

The annotated gene information of patients with ACLF and healthy subjects in GSE139602 was uploaded to the GSEA software for holistic analysis. Pathways with |NES|≥ 1.0 and NOM *p*-value < 0.05 were considered significantly enriched gene sets. Graupera’s research showed an upregulation of inflammation, fibrosis, and apoptosis networks throughout disease progression in the GSEA of the defined processes. Our GSEA analysis also found inflammation and apoptosis networks. Moreover, the GSEA results revealed significant enrichment in RCD and immune signaling pathways, including pyroptosis, apoptosis, NOD-like receptor signaling pathway, and necroptosis and regulated necrosis (Fig. 3A,B). We screened out PANoptosis-related 26 DEGs from the intersection of pyroptosis, apoptosis, necroptosis, and DEGs (Fig. 3C), and the one intersection gene was BAX. Figure 3D shows the heat map of these 26 genes, and Fig. 3E presents the results of correlation analysis of these genes.

### PPI network of DEGs and correlation analysis of immune infiltration and gene modules

**Figure 4:**
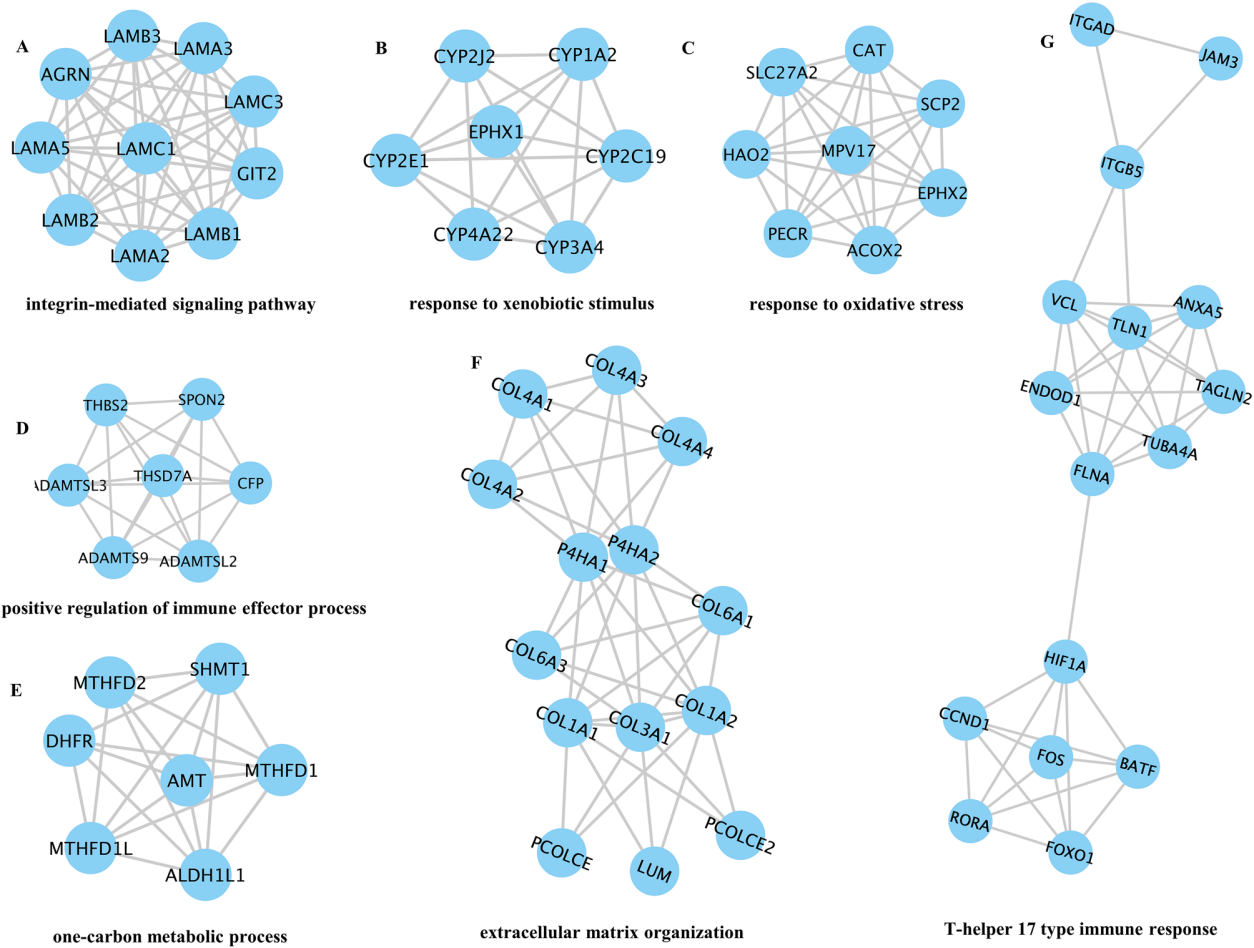
Seven clusters in the PPI network.

**Figure 5:**
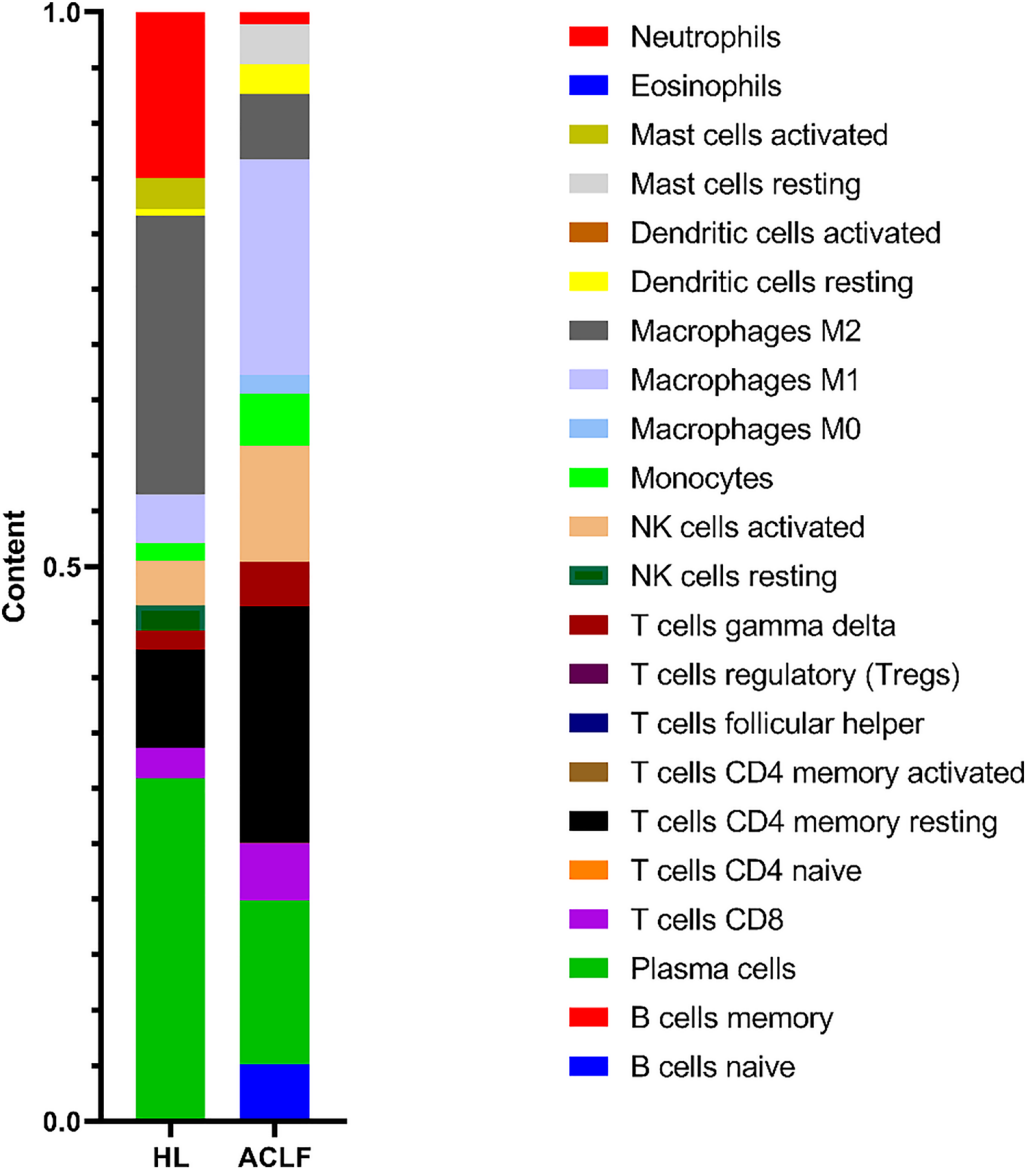
Immune cell composition in ACLF. Proportions of 22 immune cells in ACLF patients and HL.

We imported 1446 DEGs into Cytoscape. Then, the PPI network was constructed, resulting in seven gene clusters (Fig. 4). The genes contained in the first subnetwork were LAMB3, LAMA3, ACRN, LAMC3, LAMA, LAMCI, GIT2, LAMB2, LAMB1, and LAMA2 (Fig. 4A), those genes were related to the integrin-mediated signaling pathway. The genes contained in the second subnetwork were CYP2J2, CYP1A2, EPHX1, CYP2E1, CYP2C19, CYP4A22, and CYP3A4 (Fig. 4B), those genes were related with the response to xenobiotic stimulus. The genes contained in the third subnetwork were CAT, SLC27A2, SCP2, HAO2, MPV17, EPHX2, PECR, and ACOX2 (Fig. 4C), those genes were related to the response to oxidative stress. The genes contained in the fourth subnetwork were THBS2, SPON2, DAMTSL3, THSD7A, CFP, ADAMTS9, and ADAMTSL2 (Fig. 4D), those genes were related to the positive regulation of the immune effector process. The genes contained in the fifth subnetwork were MTHFD2, SHMT1, DHFR, AMT, MTHFD1, MTHFD1L, and ALDH1L1 (Fig. 4E), those genes were related to the one-carbon metabolic process. The genes contained in the sixth subnetwork were COL4A3, COL4A4, COL4A1, COL4A2, P4HA2, P4HA1, COL6A1, COL6A3, COL1A2, COL3A1, COLIAI, PCOLCE2, LUM, and PCOLCE (Fig. 4F), those genes were related to the extracellular matrix organization. The genes contained in the seventh subnetwork were ITGAD, JAM3, TGBS, ANXAS, VCL, TLNI, TAGLN2, ENDOD, TUBAA, FLNA, HIFIA, CCNDI, BATF, RORA, FOXO1, and FOS (Fig. 4G), those genes were related to the T-helper 17 type immune response. We used the CIBERSORT algorithm to investigate the correlation between immune infiltration and gene coexpression modules. The proportion of each immune cell type in each sample is shown in a stacked bar graph (Fig. 5). We obtained 22 types of immune cells, and *P* < 0.05 was considered a significant difference. The results showed that the content of Dendritic cells resting, Macrophages M0, Macrophages M1, Monocytes, NK cells activated, T cells gamma delta, T cells CD4 memory resting, T cells CD8, and B cells naive were high in ACLF.

### PANoptosis occur in liver failure

We included 187 articles in this study, including 19 on pyroptosis, 151 on apoptosis, and 17 on necroptosis. Further, 6 studies were on patients with liver failure, 93 studies were on rodents (including rats and mice), 14 studies were on primary cultured cells or cell lines, and 76 studies were on rodents and cells. Most researchers used paracetamol (APAP), LPS, D-GalN, CCL4, TNF-α, TGF-β1, and multiplicity of infection(MOI) to induce the liver failure model. The majorly used cell lines included primary hepatocytes (human, mouse, and rat), HepG2, L02, HL-7702, BNLCL2, Hepa1-6, Hep3B, AML12, BRL-3A, PHH, L929, Huh-7, Chang Liver, Hepa1, NMH, IHH, HepaRG, and miHeps. Supplementary Table S1 shows the collated information on cell types, intervention methods, cell death types, evaluation methods, and detection of key proteins in these studies. Most of the animal experiments were performed on rodents. Liver failure was induced by hepatotoxic drugs, such as APAP, LPS, D-GalN, CCL4, human serum albumin (HSA), concanavalin-A, and TNF, or surgical simulation including cecal ligation puncture, partial hepatectomy, and bile duct ligation. Some authors induced the liver failure model using viral infection (HBsAg). Sprague–Dawley, Wistar, and F344 rats and C57BL/6, BALB/c, CD-1, ICR, Kunming, and CD-1 mice were used for establishing liver failure models. Supplementary Tables S2 and S3 list the animal types, modeling methods, cell death types, evaluation methods, and key proteins detected in the experiments. The primary type of liver failure was ACLF in the studies on patients with liver failure. Supplementary Table S4 presents the cell death types, evaluation methods, key proteins detected in human experiments. All these methods to establish experimental animal models of liver failure have been adequately standardized in this research field. Our findings revealed that three types of RCD (pyroptosis, apoptosis, and necroptosis) may occur concurrently in the same cell model or animal disease model, indicating that PANoptosis occurs in liver failure.

### Histologic findings

**Figure 6:**
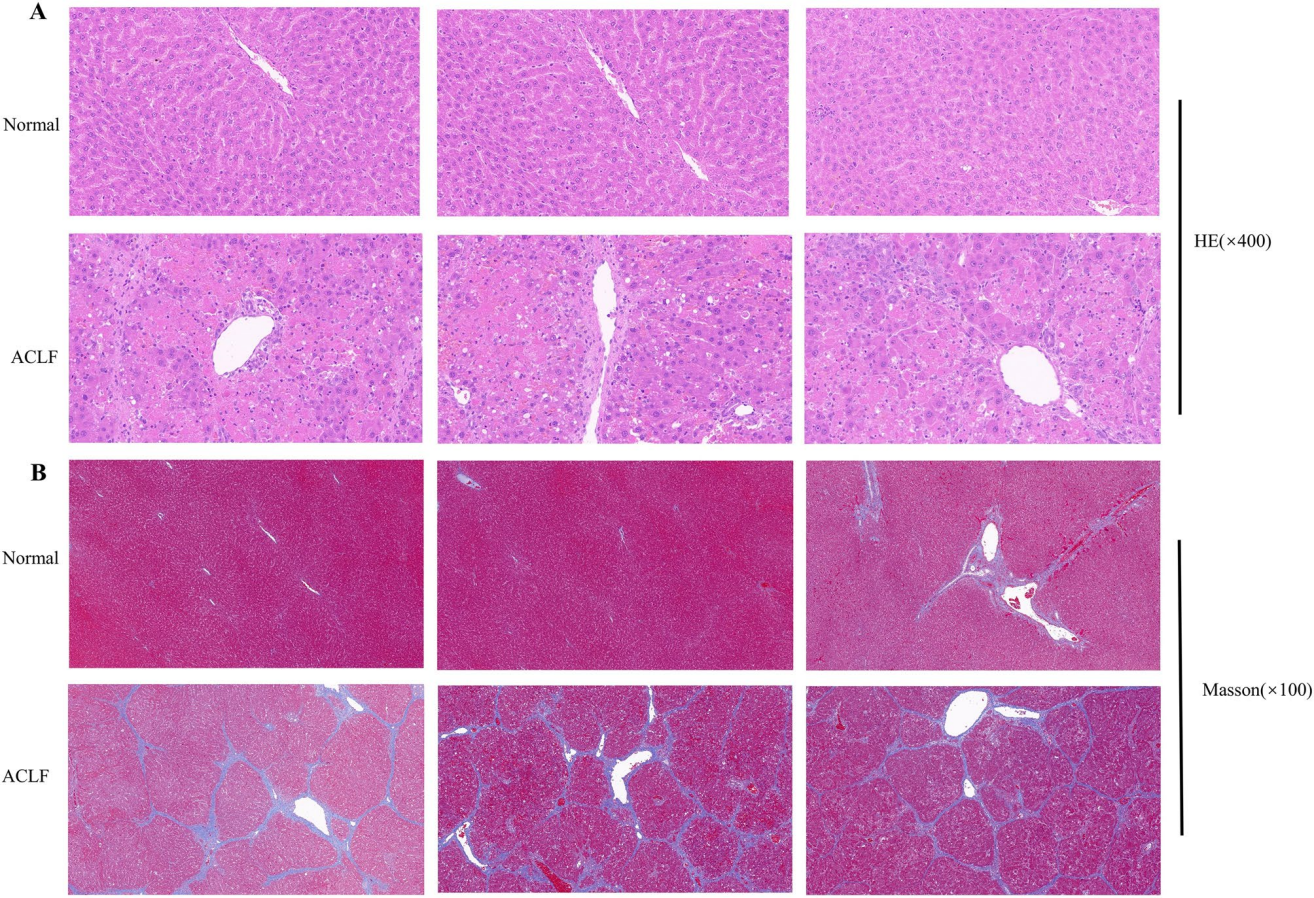
Comparison of liver tissue histopathology in normal and ACLF groups. (**A**) HE staining of hepatic tissues from the normal and ACLF group (magnification: × 400; 20 µm). (**B**) Masson staining of hepatic tissues from the normal and ACLF group (magnification: × 100; 100 µm).

The results of HE staining indicated that the structure of the liver lobule of the normal rats was clear and complete, and the hepatocytes were orderly arranged without degeneration, necrotic cells, and apparent inflammatory cell infiltration. However, in the ACLF group, the hepatocytes were disordered and necrotic accompanied by numerous infiltrated inflammatory cells, evident hepatic sinusoid expansion and hemorrhage, and massive and sub-massive necrosis of the liver tissue (Fig. 6A). The results of Masson staining showed that the morphology and structure of the hepatic lobule and portal area of rats in the normal group were complete, and the hepatic cords were arranged radially. In the ACLF group, the collagen in most portal areas in the liver tissue of rats was markedly proliferative, and some portal areas were bridged with proliferative collagen fibers. The structure of hepatic lobules was destroyed, pseudolobules were widely formed, and liver fibrosis and cirrhosis were prominent (Fig. 6B).

### Biochemical parameters and inflammatory factors in the sera of rats

**Figure 7:**
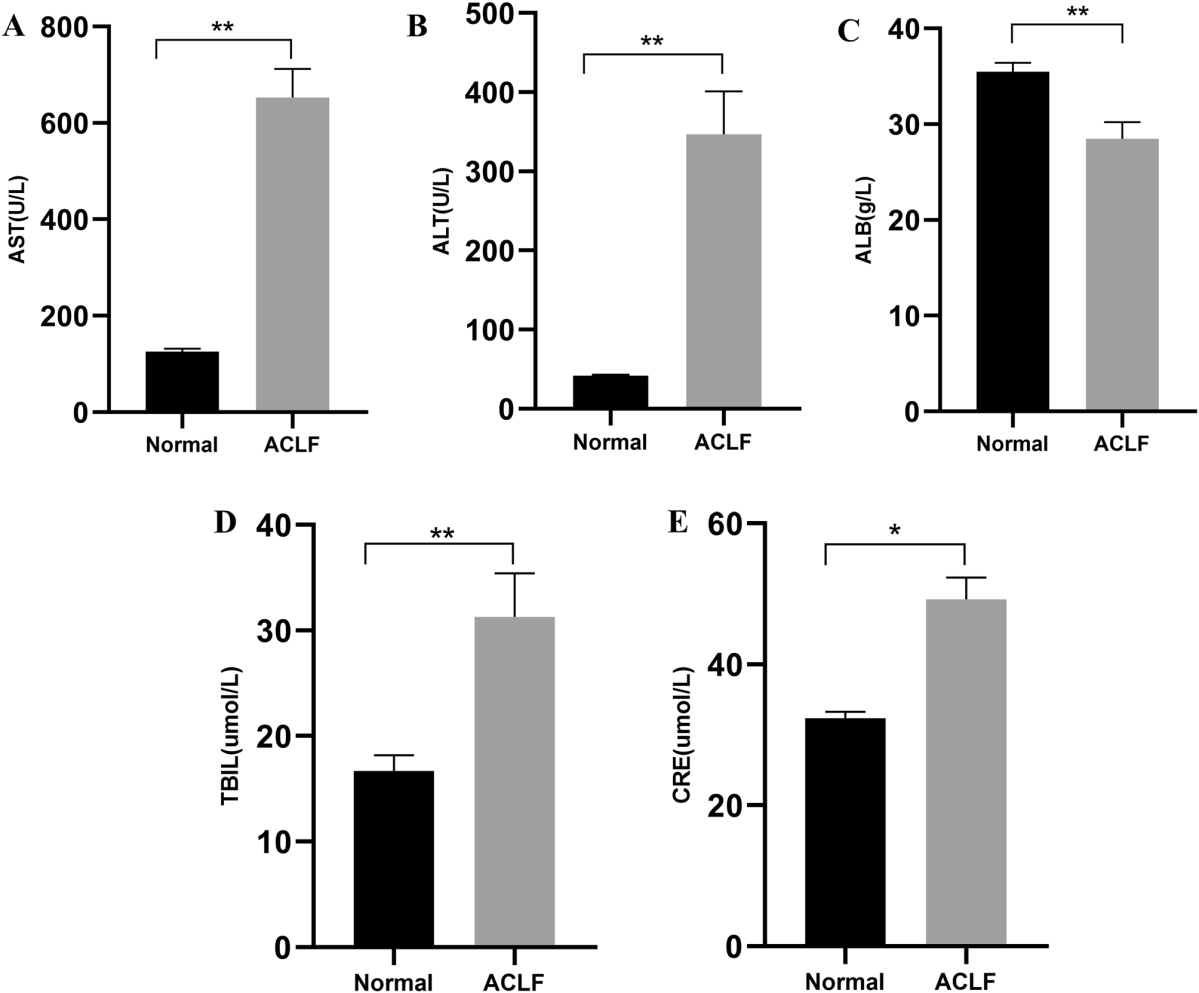
Liver and renal function of rats in normal and ACLF groups. Serum concentrations of (**A**) aspartate aminotransferase (AST) (**B**) aspartate aminotransferase (AST) (**C**) total bilirubin (TBIL) (**D**) albumin (ALB) (**E**) creatinine (CRE). Compared with the normal group, *P < 0.05 and **P < 0.01. Normal, n = 9, ACLF, n = 11.

**Figure 8:**
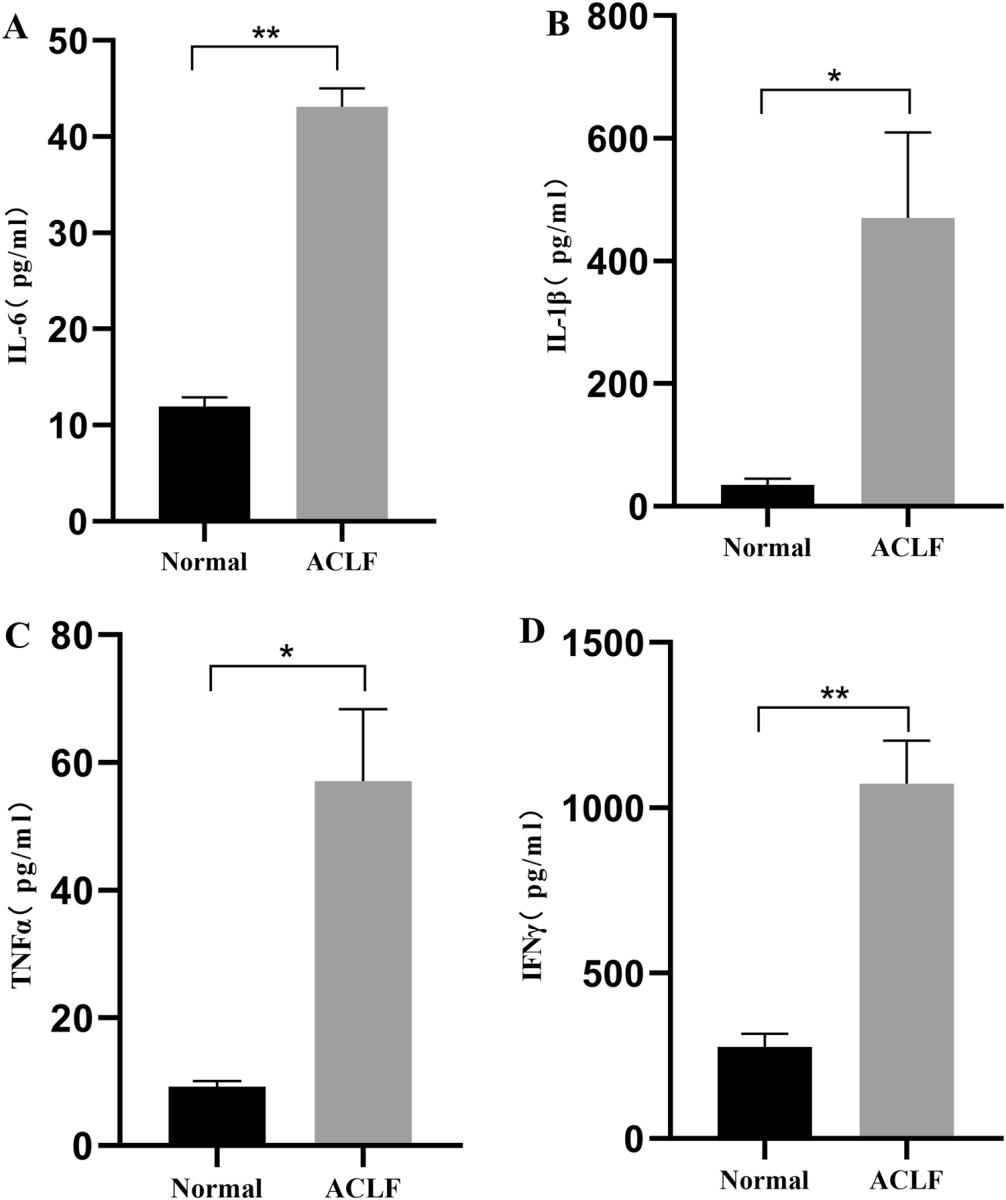
Serum concentrations of inflammatory factor in normal and ACLF group rats. (**A**) IL-6 (**B**) TNFα (**C**) IFNγ (**C**) IL-1β. Compared with the normal group, *P < 0.05 and **P < 0.01. Normal, n = 9, ACLF, n = 11.

We detected the serum ALT, AST, TBIL, ALB, and CRE concentrations to observe the liver and kidney function of rats in each group. ALT, AST, TBIL, and CRE concentrations in the ACLF group were significantly higher than those in the normal group (*P* < 0.05; *P* < 0.01). The serum ALB concentration in the ACLF group was significantly lower than that in the normal group (P < 0.01; Fig. 7A–E). ELISA results suggested that the serum concentrations of IL-6, IL-1β, IFNγ, and TNFα in the ACLF group were significantly higher than those in the normal group (*P* < 0.05; *P* < 0.01; Fig. 8A–D).

### Expression of the key proteins in PANoptosis-like cell death in ACLF

**Figure 9:**
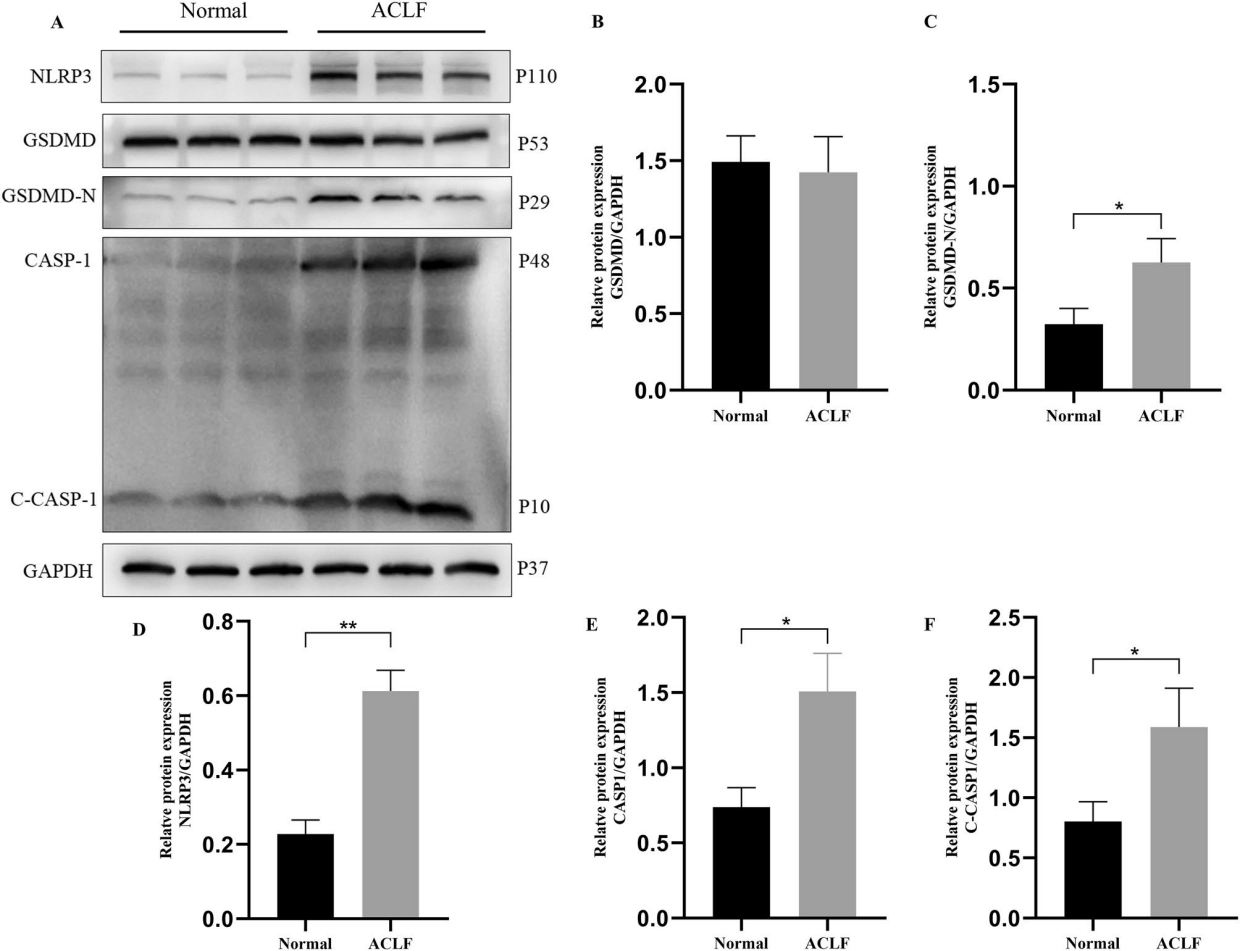
Key proteins of pyroptosis are upregulated in ACLF rats. (**A**) Western blot analysis for CASP1, C-CASP1, NLRP3, GSDMD, GSDMD-N, and GAPDH proteins. Normal group: Lanes 1 to 3, ACLF group: Lanes 4 to 6. Relative expression levels of (**B**) GSDMD/GAPDH (**C**) GSDMD-N/GAPDH (**D**) NLRP3/GAPDH (**E**) CASP1/GAPDH (**F**) C-CASP1/GAPDH. *P < 0.05 and **P < 0.01. n = 3 per group.

**Figure 10:**
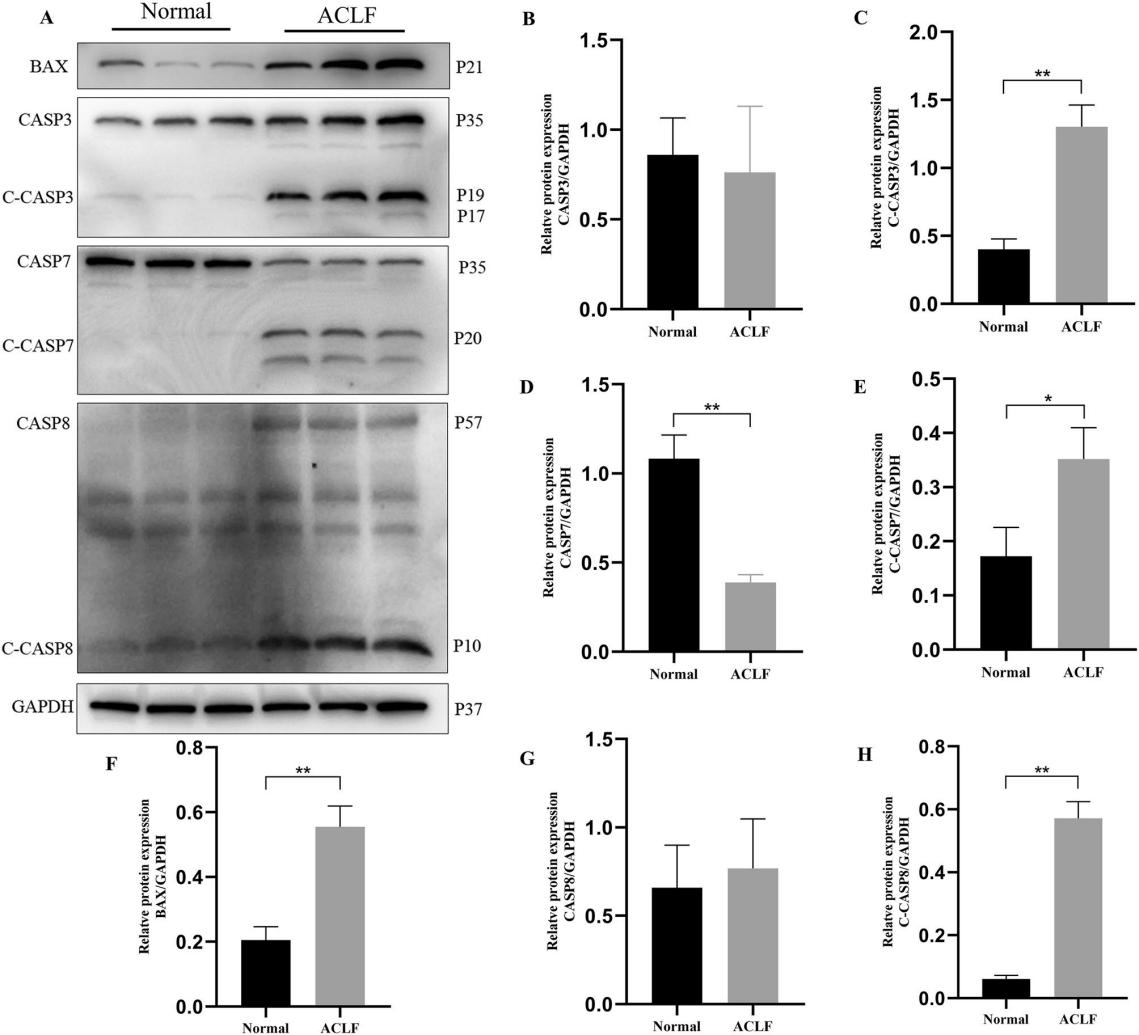
Key proteins of apoptosis are upregulated in ACLF rats. (**A**) Western blot analysis for the BAX, CASP3, C-CASP3, CASP7, C-CASP7, CASP8, C-CASP8, and GAPDH proteins. Normal group: Lanes 1 to 3, ACLF group: Lanes 4 to 6. Relative expression level of (**B**) CASP3/GAPDH (**C**) C-CASP3/GAPDH (**D**) CASP7/GAPDH (**E**) C-CASP7/GAPDH (**F**) BAX/GAPDH (**G**) CASP8/GAPDH (**H**) C-CASP8/GAPDH. *P < 0.05 and **P < 0.01. n = 3 per group.

**Figure 11:**
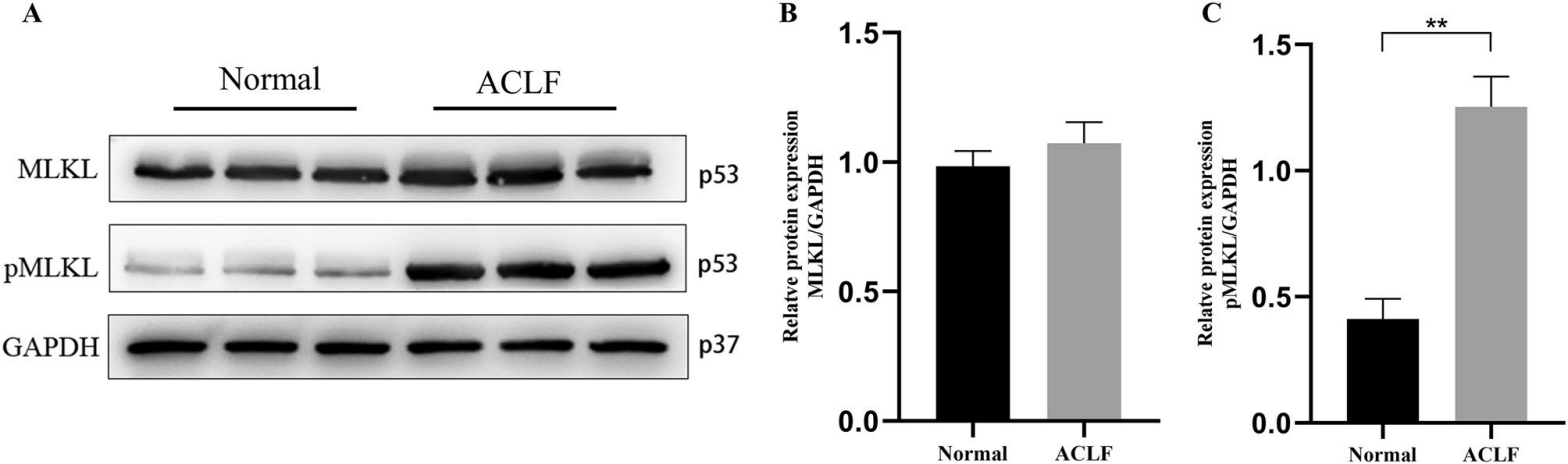
Key proteins of necroptosis are upregulated in ACLF rats. (**A**) Western blot analysis for the MLKL, phosphorylated MLKL, and GAPDH proteins. Normal group: Lanes 1 to 3, ACLF group: Lanes 4 to 6. Relative expression level of (**B**) MLKL /GAPDH. (**C**) pMLKL/GAPDH. * *P* < 0.05, * * P < 0.01. n = 3 per group.

We detected the expression of PANoptosis-associated key proteins in the liver of ACLF rats using western blotting to observe whether PANoptosis-like cell death was activated and involved in the progression of ACLF. The ACLF group showed an increased expression of NLRP3 and cleaved caspase-1 (C-CASP-1, p10 peptide). The expression of cleaved GSDMD (GSDMD-N, p29 peptide) was also upregulated in the ACLF group (Fig. 9A–F). The expression of BAX, cleaved CASP3(C-CASP-3, p19 peptide, p17 peptide), CASP7(C-CASP-7, p20 peptide), and CASP8(C-CASP-8, p10 peptide) were notably increased (Fig. 10A–H). Figure 11A–C suggests that the expression of phospho-MLKL was significantly upregulated in the ACLF group. Overall, these results indicated that the key proteins of pyroptosis, apoptosis, and necroptosis were expressed in the rat liver tissues from the ACLF group, thereby indicating the occurrence of PANoptosis in ACLF.

### Co-treatment of TNFα and IFN γ induces PANoptosis in L02 cells

**Figure 12:**
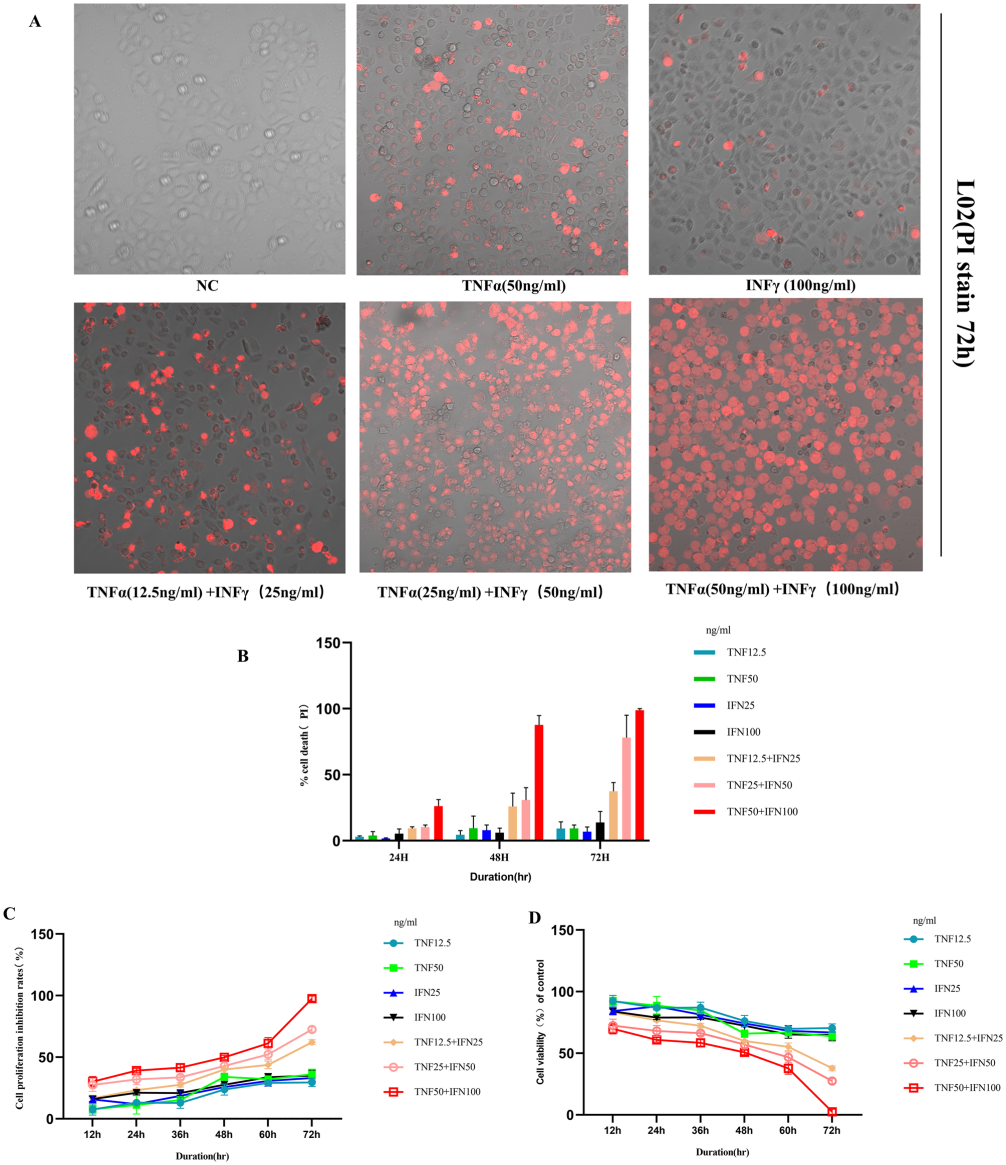
Co-treatment of TNF-α and IFN-γ Induces Cell Death. (**A**) Representative images of cell death in L02 after 72 h of the indicated treatments, measured by PI stain. Red indicates dead cells. Scale bar, 50 μm. (**B**) Quantification of the cell death in (**A**). (**C**–**D**). L02 cells were stimulated with the following concentrations of cytokines, TNF-α (12.5, 25, and 50 ng/ml)/IFN-γ (25, 50, and 100 ng/ml) for 12, 24, 36, 48, 60, and 72 h, measured by CCK8.

Our results show that TNFα and IFN γ might synergize to induce cell death. However, the addition of either TNFα or IFN γ individually still failed to induce cell death, further supporting that synergism between TNFα and IFN γ is critical in inducing cell death. The dynamics of cell death could be affected by the concentration of cytokines chosen. To confirm that the observed cell death was not an artifact of the cytokine concentration, we analyzed the effect of changing the concentrations of the cytokines and found that increasing the concentration up to threefold still failed to induce cell death. Conversely, the kinetics of cell death induced by TNFα and IFN γ co-stimulation were proportional to the concentrations of TNFα and IFN γ (Fig. 12).

## Discussion

The systemic hyperinflammatory state is the main pathologic feature of tissue damage and organ failure in patients with ACLF^35^. According to a large, multicenter, prospective clinical observational study of 1383 consecutively hospitalized patients with acute decompensation of liver cirrhosis published by the Working Group on Chronic Liver Failure of the European Liver Society, white blood cell counts and serum C-reactive protein concentrations in patients with ACLF are significantly higher than those in patients without ACLF. These parameters are positively correlated with the number of organ failures and the severity of the disease in patients with ACLF^1^. Therefore, this Working Group proposed the hypothesis of systemic inflammatory response in ACLF as early as 2015, opening up a new direction for the research on the pathophysiology of ACLF^36^. Uncontrolled systemic inflammation induces cell death in numerous hepatic cells. Hyperactivity of the immune system may be the primary driver of rapid hepatic cell death in the early stage^12^. Cell dysfunction and death caused by excessive inflammatory response are often mediated by pro-inflammatory cytokines, such as TNFα, IL-1β, IL-6, and INFγ^13,14^. In ACLF, Kupffer cells produce a large amount of TNFα, IL-1, and IFN γ. Inflammatory factors can cause rapid liver cell death and liver tissue damage^13^, and NK/NKT cells can mediate hepatocyte death through the Fas/FasL system and NKG2D/NKG2DL signaling pathway. Cytotoxic T cells can also mediate rapid hepatocyte death through perforin-1/granzyme, Fas/FasL, TNFR1/TNF, and RIPK1/RIPK3 signaling pathways^37^. Th17 cells are involved in hepatocyte death and inflammatory damage by secreting a critical inflammatory factor IL-17, thereby enhancing TNF function and cell adhesion molecule expression^38^. Cytokines, such as IFNγ, TNFα, and IL-6, are closely related to hepatocyte necrosis and prognosis in patients with ACLF^15^. Two distinct immune-related pathophysiologic features are present in patients with ACLF: an excessive systemic inflammatory response and an increased susceptibility to infection because of immunosuppression^11^. ACLF is an innate immune dysfunction syndrome, and patients with ACLF show evidence of a pro-inflammatory state, including local liver inflammation, systemic inflammatory response syndrome, and vascular endothelial dysfunction. These inflammatory factors characteristically drive the progression of multi-organ failure. Moreover, the patients are immunosuppressed and show acquired immunodeficiency, making them highly susceptible to infection. Therefore, patients with ACLF have a poor prognosis along with complications, such as infections, leading to high morbidity and mortality. Cytokines and chemokines are crucial in the recruitment and activation of leukocytes in the liver and may permanently damage the liver by prolonging inflammatory pathways and damaging other organs^39^. Systemic inflammation, the main pathologic feature of ACLF, often causes irreversible liver parenchymal damage, and a storm of hyperimmune functions and inflammatory factors triggers a "domino effect" of rapid hepatocyte death, leading to the collapse of the internal hepatic ecosystem. Therefore, focusing on systemic inflammation-induced liver injury is the key to understanding the pathogenesis of ACLF, which is crucial for clinical drug development.

RCD is a genetically encoded and actively regulated cellular process for the targeted removal of redundant, irreversibly damaged, and potentially harmful cells^40^. Apoptosis, pyroptosis, and necroptosis are distinct processes that play important roles in development and limiting infections in organisms. RCD pathways operate in parallel with some degree of overlap, and pyroptosis, apoptosis, and necroptosis are closely related pathways that can be cross-regulated. PANoptosis was first described by Malireddi in 2019^18^. When PRRs recognize PAMPs expressed by microorganisms and DAMPs released after aseptic injury, they bind to inflammatory factors, transmit signals through receptors that contain death domains, and initiate a highly interconnected process of cell death known as PANoptosis. It is a unique inflammatory regulated cell death regulated by PANoptosome. PANoptosome provides a molecular scaffold for key molecules involved simultaneously in pyroptosis, apoptosis, and necroptosis. These molecules include NLRP3, CASP1, and GSDMD (for pyroptosis), CASP8, CASP3, and CASP7 (for apoptosis), and CASP8, RIPK1, RIPK3, and MLKL (for necroptosis)^41^. The simultaneous presence of these components allows the interaction and activation of multiple cell death pathways, which cannot be blocked by the end effectors of individual pathways^19^. The core proteins of the PANoptosome promote the activation of downstream cell death effector molecules, and the cytokines and DAMP initiate and enhance inflammatory responses^20^. Activation of CASP8 or CASP9 in the PANoptosome induces apoptosis, which leads to activation of the executive caspases, including CASP3 and CASP7^42,43^. Apoptotic CASP3 can also activate gasdermin E to induce the lytic form of cell death^44^. During necroptosis, proteins containing RIP homotypic interaction motifs, including RIPK1 and RIPK3, play a key role in the phosphorylation of MLKL to execute cell death. In addition, identifying the entire apical sensor that controls PANoptosis is very important for regulating this process. Sensor molecules can initiate the formation of protein complexes of PANoptosome. The differential expression and activation status of cell death signaling proteins may determine the relative extent to which each cell death pathway is activated depending on cell type and the degree of inflammatory stimulation. Currently, the sensors have been identified as Z-DNA binding protein 1 (ZBP1) and TGFβ activated kinase-1 (TAK1)^18^. ZBP1 is an innate immune receptor that senses nucleic acids and activates PANoptosis and inflammation. Its activation leads to the recruitment of RIPK3 and CASP8 which interact with its receptor to form a cell death signaling scaffold. The ZBP1–RIPK3–CASP8 complex is involved in NLRP3 inflammatory vesicle-dependent focal death, CASP8-mediated apoptosis, and RIPK3/MLKL-driven necroptosis^18^. Genetic deficiency or microbial or pharmacologic inactivation of TAK1 function triggers RIPK1-dependent PANoptosis cell death complex assembly. The PANoptosis cell death complex containing RIPK1, FADD, and CASP-8 acts as the core. This complex is involved in CASP8-mediated apoptosis, and inhibition of CASP8 activity promotes RIPK3/MLKL-dependent necroptosis^18^. PANoptosis has been observed in infections, autoinflammatory diseases, and cancers ^45^. These diseases involve the recognition of PAMPs and DAMPs by PRRs, which are involved in driving intracellular signaling cascades that ultimately lead to the transcription and synthesis of inflammatory mediators. A classic example is the binding of TLR4 to LPS, a PAMP from the cell wall of gram-negative bacteria, leading to downstream transcription and activation of multiple inflammatory mediators and cytokines^46^. ACLF involves reduced baseline liver reserve and long-term circulatory dysfunction; therefore, the initiating event may be triggered by hepatocyte death and the release of DAMPs because of hepatocyte necrosis. Liver-resident Kupffer cells highly express various DAMP receptors (e.g., P2X7, TLR4, TLR9, and RAGE), thereby mediating the initial response to injury^47^. Hepatocyte oxidative stress, direct mitochondrial damage, cell scorching, apoptosis, and necroptosis, result in the release of DAMPs recognizable by Kupffer cells. The activated Kupffer cells secrete pro-inflammatory cytokines, reactive oxygen species, and chemokines, amplifying pro-inflammatory signals^48,49^. PANoptosome-driven cell death and inflammatory responses are highly consistent with the strong inflammatory pathogenesis observed in ACLF. When PRRs recognize microbially expressed PAMP molecules and DAMP molecules released by aseptic injury, TLR initiation triggers inflammasome activation, including CASP-8 and GSDMD activation, and recruitment of NLRP3 and ASC into a novel RIPK1 kinase activity-independent cell death complex, which initiates PANoptosis through a receptor-conducted signal containing the death domain. The PANoptosome promotes the activation of downstream cell death effector molecules. Downstream cytokines, reactive oxygen species, chemokines, and DAMP act as alarm proteins, initiating and enhancing the inflammatory response^20^. The characteristics of PANoptosis are also at the core of understanding cytokine storms from a molecular perspective^50^. PANoptosome can fundamentally integrate cell death patterns, driving powerful forms of inflammatory cell death under pathologic conditions, which is highly consistent with the strong inflammatory pathogenesis of ACLF.

Graupera^25^ reproduced the key conclusions of patients with ACLF had significantly different gene expression in all LDRNs: oxidative stress and genetic factors were downregulated compared to those of healthy individuals, whereas inflammation, apoptosis of hepatocytes and HSCs, angiogenesis, fibrosis and resolution, senescence, and carcinogenesis networks were upregulated compared to patients with early-CLD, CC, and DC and to healthy individuals. We used biometric analysis to evaluate the potential pathways and biological processes of ACLF. In the GSE139602 dataset, we conducted enrichment analysis on 1446 DEGs and explored their interactions. GSEA is characterized by analyzing gene sets rather than individual genes, which helps to avoid the inability to reproduce a single high-score gene due to poor annotation. GSEA indicated that RCD and immunity signaling pathways were the essential pathways in ACLF, including pyroptosis, apoptosis, NOD-like receptor signaling pathway, necroptosis, and regulated necrosis. We obtained 26 genes from the intersection of pyroptosis, apoptosis, necroptosis, and DEGs to screen out PANoptosis-related DEGs. and the one intersection gene was BAX. Then, we found that upregulated DEGs were mainly involved in immune and inflammatory responses, whereas downregulated DEGs were mainly involved in regulating biosynthetic and metabolic pathways. The upregulated DEGs were mainly engaged in focal adhesion, PI3K-Akt signaling pathway, human papillomavirus infection, ECM-receptor interaction, and pathogenic *Escherichia coli* infection. Downregulated DEGs were mainly engaged in the biosynthesis of cofactors, carbon metabolism, drug metabolism-cytochrome P450, and peroxisome bile secretion. These results reflect two main biological processes that occur together during the progression of ACLF regulated by different genes: immune inflammatory response imbalance and energy metabolism. We found that pyroptosis, apoptosis, and necroptosis signaling pathways occupy essential positions in ACLF patients, and they have genes that intersect with each other. To support the hypothesis that PANoptosis plays a crucial role in liver injury associated with ACLF, it is necessary to verify the simultaneous occurrence of pyroptosis, apoptosis, and necroptosis in the existing literature on ACLF. The identification of a PANoptosome in ACLF liver injury is crucial as it is responsible for initiating the three forms of RCD simultaneously. According to recent research, a PANoptosome consists of three essential proteins: (1) ZBP1, an NLRP3 acting as a sensor, (2) an apoptosis-associated speck-like protein that contains a caspase recruit domain and a Fas-associated protein with death domain acting as complex adapters and (3) RIP1, RIP3, CASP-1, and CASP-8 that exert catalytic effects^20,45,51^. Although PANoptosome studies have been extensively conducted in relation to infectious diseases and cancer, there is a notable absence of research on PANoptosomes in the context of liver injury associated with ACLF. Our findings indicate that nucleotide-binding domain and leucine-rich repeat pyrin-domain containing protein 3, CASP-1, and apoptosis-associated speck-like protein containing a caspase recruit domain related to pyroptosis, CASP-8 and Fas-associated protein with death domain related to apoptosis, RIP1, and RIP3 related to necroptosis have all been identified as marker proteins in animal models and/or cell models of LF (Supplementary Tables S1–S4).Our literature mining data suggested that three forms of cell death can concurrently occur in liver failure. Further, their molecular mechanisms have inflammation-related parts^34,52,53^. These findings overlap with the inflammatory and immune-related aspects of existing studies on PANoptosis. We found that three types of RCD may occur simultaneously in the same liver failure cell or animal model, indicating that PANoptosis may exist in liver failure. This suggests the possibility of PANoptosis in ACLF at the pathological mechanism level. Our primary purpose was to investigate whether PANoptosis-like cell death exists in ACLF and whether it plays a role in liver injury and failure. We selected Wistar rats for in vivo experiments and used CCL4 combined with d-galactosamine and LPS to construct the ACLF model. Our experimental data revealed that liver and renal dysfunction in ACLF rats was accompanied by the extensive release of inflammatory factors. Hepatocytes were disordered and necrotic, accompanied by a significant number of inflammatory cell infiltration, noticeable hepatic sinusoid expansion and hemorrhage, and massive and submassive necrosis of liver tissue. Moreover, the structure of hepatic lobules was destroyed, pseudolobules were widely formed, and liver fibrosis and cirrhosis were prominent. We detected the expression of key proteins of PANoptosis in the liver of ACLF rats. The ACLF group showed an increased expression of NLRP3 and C-CASP1, a key protein in the pyroptosis pathway. The ACLF group also showed an upregulated expression of GSDMD-N. GSDMD cleavage produces an active p29 fragment that can form membrane pores to induce pyroptosis. GSDMD can be activated by inflammatory bodies downstream of caspase-1 to release the p29 fragment. Consistent with the production of GSDMD-N, activation of caspase-1 produces C-CASP1. The results showed apoptosis in ACLF, as evidenced by the cleavage of CASP3, CASP7, and CASP8. In addition, compared to the normal group, the expression of BAX, a key proapoptotic protein was significantly increased in the ACLF group. The increased phosphorylation level of MLKL is a marker of necroptosis. ACLF rats showed significantly upregulated expression of phospho-MLKL. *Cell* reported in 2020 that TNF-α/INF-γ could induce murine BMDM, human monocyte cell line THP-1, primary human umbilical cord venous endothelial cells to undergo massive PANoptosis, and blocking TNFα/IFNγ could reverse cell death. However, it was found that other cytokines could not have the same effect, and it was also verified in animal models of sepsis, severe neo-coronary patients (combined with multiple organ failure), and cytokine-induced shock^22^. Inflammation levels in patients with ACLF are not comparable to those of patients with sepsis, and it has been found that PANoptosis exists in sepsis^54^. Moreover, a large body of literature confirms that TNF-α and INF-γ are highly expressed in ACLF patients and are closely related to hepatocyte necrosis and prognosis in ACLF patients. Based on this, we hypothesized that combining TNF-α/INF-γ inflammatory cytokines could induce hepatocellular PANoptosis, and our experimental results confirmed this. Our in vitro and in vivo experimental data showed pyroptosis, apoptosis, and necroptosis occurred simultaneously after liver injury of ACLF in rats and L02 cells injury induced by TNFα/IFNγ. These data support the existence of PANoptosis, indicating the presence of PANoptosis-like cell death in the liver injury of ACLF. All of these proteins are considered components of PANoptosome. Although there are no studies on the components of PANoptosome involved in liver injury or failure related to assembly, the existing data suggest that the "raw materials" that make up PANoptosome are highly expressed in liver failure.

However, we summarized the preliminary studies on PANoptosis to characterize the presence of PANoptosis-like cell death in liver failure. Our future experimental studies will focus on PANoptosome in liver injury caused by ACLF and the critical molecules used to regulate PANoptosome. Overall, a better understanding of the molecular basis of PANoptosis can provide vital information for developing targeted inhibitors and activators for regulating inflammation and immune responses and treating inflammatory cell death.

## Conclusion

Evidence from literature mining, bioinformatic analyses, and animal model studies suggest that PANoptosis may play a more significant role than anticipated in ACLF and is critical for controlling hepatocyte death. Our results indicate new opportunities for designing potential therapeutic interventions for ACLF. However, further studies are needed to determine the underlying molecular mechanisms.

### Supplementary Information

Supplementary Figures.

Supplementary Table S1.

Supplementary Table S2.

Supplementary Table S3.

Supplementary Table S4.

## Data Availability

The data used to support the findings of this study are available from the corresponding author upon reasonable request.
