# Supplementary material for: PANoptosis-like death in acute-on-chronic liver failure injury
Source: Sci Rep. 2024 Jan 3;14:392. doi: 10.1038/s41598-023-50720-1 (PMC10764922; doi:10.1038/s41598-023-50720-1)

NLRP3

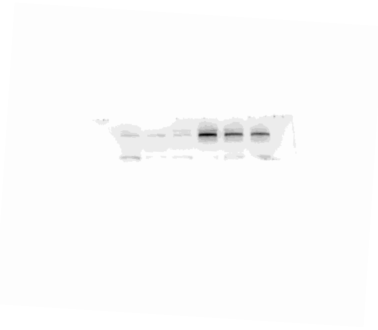

GSDMD

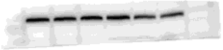

GSDMD-N

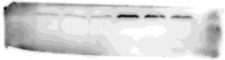

CASP-1,C-CASP-1

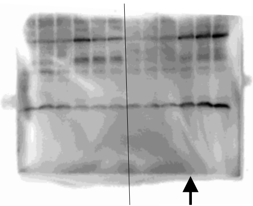

The right side of the black line is the cropped gels/blots

GAPDH

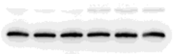

BAX

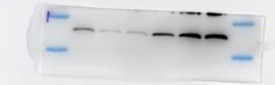

CASP-3,C-CASP-3

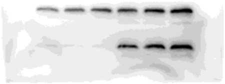

CASP-7,C-CASP-7

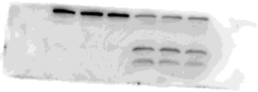

CASP-8,C-CASP-8

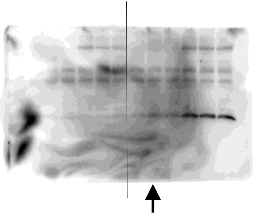

The right side of the black line is the cropped gels/blots

GAPDH

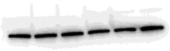

MLKL

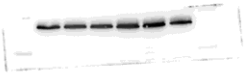

pMLKL

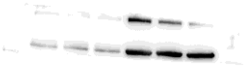

GAPDH

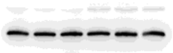

Supplement: Supplementary file 1 — Supplementary Figures. [file 41598_2023_50720_MOESM1_ESM.pdf]
